# Maladaptive behaviors in children with autism and parental hopelessness: The moderating role of parental reflective functioning

**DOI:** 10.1002/aur.2841

**Published:** 2022-11-05

**Authors:** Yael Enav, Marguerite V. Knudtson, Antonio Y. Hardan, James J. Gross

**Affiliations:** ^1^ Department of Counseling and Human Development University of Haifa Haifa Israel; ^2^ Department of Psychology Stanford University Stanford California USA

**Keywords:** adults, affect/emotion, children, clinical psychology, restricted/repetitive behaviors

## Abstract

Hopelessness in parents has implications for parents' own well‐being as well as their ability to meet the needs of their children. In the present study, we examined the effect of maladaptive behaviors in children with autism on parental hopelessness, with particular attention to whether parental reflective functioning would moderate the effect of maladaptive behaviors on parental hopelessness. Our sample included 68 parents of children with autism between the ages of 3 and 18. Findings revealed a significant positive relationship between maladaptive behaviors in the children and hopelessness in the parents. Moreover, parental reflective functioning moderated the effect of child maladaptive behaviors on parental hopelessness, such that children's maladaptive behaviors were positively associated with parental hopelessness in parents with low (but not high) reflective functioning. Findings suggest parental reflective functioning may be a protective factor against parental hopelessness, and thus a possible target for interventions for hopelessness in parents whose children with autism exhibit greater maladaptive behaviors.

## Lay Summary

Parental hopelessness is important for parents and children well‐being. In the present study, we examined the effect of maladaptive behaviors in children with autism on parental hopelessness, with paying attention to parental reflectivity and its moderating role. Our sample included 68 parents of children with autism between the ages of 3 and 18. Findings showed that greater maladaptive behaviors in the children are related to greater hopelessness in the parents but this relationship was only for parents with low reflective capabilities and not for parents with high reflective capabilities. Our findings propose that parental reflective capabilities may protect parents from hopelessness.

## INTRODUCTION

Hopelessness is characterized by negative expectations regarding oneself and one's future (Beck et al., 1974). Hopelessness has received a great deal of attention over the past several decades because of its important role in predicting depression and other disorders. It is therefore concerning that hopelessness appears to be elevated in parents of children with autism (Naci & Koletsi, 2021). In this paper, we consider whether children's maladaptive behaviors predict parental hopelessness, and if so, whether parental reflectiveness might moderate these effects.

## CHILDREN'S MALADAPTIVE BEHAVIORS AND PARENTAL HOPELESSNESS

Maladaptive behaviors are defined as behaviors that interfere with everyday life and have the potential to cause harm to those exhibiting the behaviors and others around them (Gray, 2013). The two major types of maladaptive behaviors include externalizing behaviors, such as tantrums, aggression and self‐injury (Kauten & Barry, 2020), and internalizing behaviors, such as fear, withdrawal and anxiety (Gresham et al., 1999).

Maladaptive behavior is known to be a predictor of parental negative affect and ill‐being in parents of children with autism (Enea & Rusu, 2020), but there is no evidence that maladaptive behavior is a predictor of hopelessness in parents of children with autism. However, studies have revealed a positive relationship between externalizing behaviors in children and hopelessness in their parents in other populations, such as parents of juvenile offenders (Bradshaw et al., 2006) and parents of children with intellectual disabilities (Padencheri & Russell, 2002). Our aim here is to fill out the gap in parents of children with autism.

## PARENTAL REFLECTIVE FUNCTIONING

One factor that may moderate the link between children's maladaptive behavior and parental hopelessness is parental reflective functioning (PRF). PRF is defined as the parent's ability to understand their child's behavior in light of their own and their child's mental states (Fonagy et al., 2006). Such an understanding might play a role in mitigating the impact of children's maladaptive behavior on parental hopelessness, and PRF would seem to be a particularly important skill in families with children with autism as parents need to work harder to be successful in this skill and often without reciprocation from their child (Slade, 2009).

Indeed, there is some evidence, which suggests that PRF may serve as a moderator for the relationship between maladaptive behaviors in children and parental mental health. For example, a recent study by Khoshroo and Seyed Mousavi (2021) with a non‐clinical sample found PRF to moderate the relationship between internalizing and externalizing symptoms in children and maternal depression such that depressed mothers with lower PRF had children with greater internalizing and externalizing symptoms than those with greater PRF. It is not yet clear, however, whether PRF might moderate the link between children's maladaptive behavior and parental hopelessness.

## THE PRESENT STUDY

The goal of the present study was to examine the relationship between maladaptive behaviors in children with autism and parental hopelessness. Based on previous studies, we hypothesized that there would be a significant positive relationship between maladaptive behaviors in children with autism and hopelessness in their parents. We expected this relationship to be moderated by PRF, such that parents low in PRF would show a stronger positive relationship between children's maladaptive behaviors and parents' hopelessness.

Furthermore, as the VABS‐II‐MBI contains three subscales, externalizing behaviors, internalizing behaviors, and other behaviors, we conducted exploratory analyses to examine the effect of each subscale on parental hopelessness.

## METHOD

The data used for the current study were collected as part of a 4‐week, non‐randomized controlled intervention designed to improve emotion regulation and reflective functioning skills in parents of children with autism (Enav et al., 2019). During the workshop, parents were presented with lectures and participated in group discussions and role‐play to develop strategies to regulate their emotions and enhance their reflective functioning related to their children's behavior and emotions. The study was approved by Stanford University's Institutional Review Board and was registered in the Clinical Trials database. All data used in the present study were obtained prior to parents' participation in the intervention. None of the predictor or outcome variables used in the present study were used in previous analyses.

### Participants

Participant recruitment occurred through distribution of fliers at clinics, schools and autism awareness events. Parents of children with autism diagnoses were included regardless of the severity or type. Parents who required a translator or other language assistance were excluded due to the clinical interview being conducted in English. Our sample included 68 parents or primary caregivers of a child between the age of 3 and 18 years with an official diagnosis of autism. Given that the researchers were interested in improving emotion regulation and reflective functioning skills spanning multiple contexts, recruitment focused on a wide child age range so as not to limit the types of behavioral challenges experienced by parents.

Participants were predominantly highly‐educated, Caucasian or Asian/Pacific Islander females who were married or in a domestic partnership. The children of the caregivers participating in the study were a majority male (74%) and tended to have lower autism severity. See Table 1 for complete demographic characteristics of the study sample.

**TABLE 1:** Demographic characteristics of study sample

Demographic factor	Frequency	Percent
Parents' sex		
Male	12	18
Female	56	82
Race		
Caucasian	34	50
Asian/Pacific Islander	21	31
Hispanic/Latino	4	6
Other	7	10
Missing	2	3
Marital status		
Married/domestic partner	64	94
Single	1	1.5
Divorced	1	1.5
Widowed	1	1.5
Missing	1	1.5
Education status		
Graduate degree	32	47
Bachelor's degree	13	19
Some college	12	18
PhD, MD, or JD	4	6
High school or GED	2	3
Missing	5	7
Employment status		
Homemaker	22	32
Part‐time	15	22
Full‐time	15	22
Self‐employed	8	12
Unemployed	1	2
Retired	2	3
Missing	5	7
ASD severity		
Low	34	50
Moderately low	5	7
Mild	9	13
Moderately high	4	6
High	3	5
Missing	13	19
Children's sex		
Male	50	74
Female	17	25
Missing	1	1

Age (years)	Range	Mean	SD
Parents	31–64	45	7.70
Children	3–18	10	4.15

Abbreviation: SD, standard deviation.

### Procedures

After expressing interest, parents were screened by phone for their availability, official diagnosis for their child with autism, and English proficiency. All participants came in for a 2–3‐h assessment session at the beginning of the study. Participants filled out a series of questionnaires, as described below. For a description of the full procedures of the original study, refer to Enav et al., 2019.

### Measures

#### Demographics

The following information was collected pertaining to the parents: age, sex, marital status, education level, and employment status. In addition, parents were asked to specify the number of children they had, the age of their child with autism, and the severity of their children's autism on a Likert Scale from 1 (Low) to 5 (High).

#### Child behavior

The Maladaptive Behavior Index of the Vineland Adaptive Behaviors Scales, Second Edition (VABS‐II‐MBI; Sparrow et al., 2005) was used to measure maladaptive behaviors in children. The VABS‐II‐MBI includes three subscales: externalizing behaviors (10 items, e.g., has temper tantrums; is physically aggressive), internalizing behaviors (11 items, ex: Is overly anxious; Avoids social interaction), and other maladaptive behaviors (15 items, e.g., acts overly familiar with strangers; has a hard time paying attention). Parents scored each item on a Likert Scale from 0 (Never) to 2 (Usually). Scores for each subscale were calculated as the sum of items for each subscale respectively. Total maladaptive behavior scores were calculated as the sum of each subscale score. Higher scores represent greater maladaptive behaviors.

#### Parental hopelessness

The Beck Hopelessness Scale (BHS; Beck et al., 1974) was used to measure parental hopelessness. The BHS is a questionnaire consisting of 20 items (e.g., I look forward to the future with hope and enthusiasm; I don't expect to get what I really want). Parents rated the degree to which they agreed with each statement using a Likert Scale from 1 (Always False) to 5 (Always True). The responses were averaged to produce a total hopelessness score. Higher scores on the BHS reflect greater levels of hopelessness in parents.

#### Parental reflective functioning

The Parental Reflective Functioning Questionnaire (PRFQ; Luyten et al., 2017) was used to assess Parental Reflective Functioning. The PRFQ is a 39‐item, parent‐completed questionnaire (e.g., “I am often curious to find out how my child feels” and “My child cries around strangers to embarrass me” that is reverse scored). Parents rated each item on a Likert Scale from 1 (Strongly Disagree) to 7 (Strongly Agree). The responses for each item were averaged to produce a total PRF score. Higher scores on the PRFQ reflect greater reflective functioning skills in parents.

### Statistical analyses

To examine the association between child maladaptive behaviors and parental hopelessness, a simple linear regression was conducted with VABS‐II‐MBI scores as the predictor variable and BHS scores as the outcome variable. To examine associations between each of the subscales of child maladaptive behavior and parental hopelessness, a simple linear regression was conducted with each of the VABS‐II‐MBI subscales (Externalizing Behavior, Internalizing Behavior, Other Behaviors) as the predictor variables and BHS scores as the outcome variable. To examine the moderation effects of PRF on the relationship between child maladaptive behaviors and parental hopelessness, PRFQ scores were used as a moderator variable in the regression of VABS‐II‐MBI scores on BHS scores. Children's age and autism severity were held as covariates and assumptions were met for each regression analysis.

## RESULTS

### Parental child maladaptive behavior and hopelessness

The simple linear regression predicting parental hopelessness, measured using the BHS, from child maladaptive behavior, measured using the VABS‐II‐MBI, indicated a significant positive effect (*F*(1,43) = 5.36, *p* < 0.05, adjusted *R* ^2^ = 0.10). Parents with higher levels of hopelessness had children with greater maladaptive behaviors. Results remained significant after controlling for children's age and autism severity.

When the relationship between parental hopelessness and each subscale of the VABS‐II‐MBI was examined separately, the Other Maladaptive Behaviors subscale emerged as significant (*F*(1,44) = 5.56, *p* < 0.05, adjusted *R* ^2^ = 0.09), while the Externalizing Behaviors (*F*[1,48] = 2.83, *p* = 0.10, adjusted *R* ^2^ = 0.04) and Internalizing Behaviors (*F*(1,48) = 3.32, *p* = 0.07, adjusted *R* ^2^ = 0.05) subscales were each contributing to the effect but did not individually reach statistical significance. All results remained significant after controlling for children's age and autism severity.

### Moderation by parental reflective functioning

**FIGURE 1:**
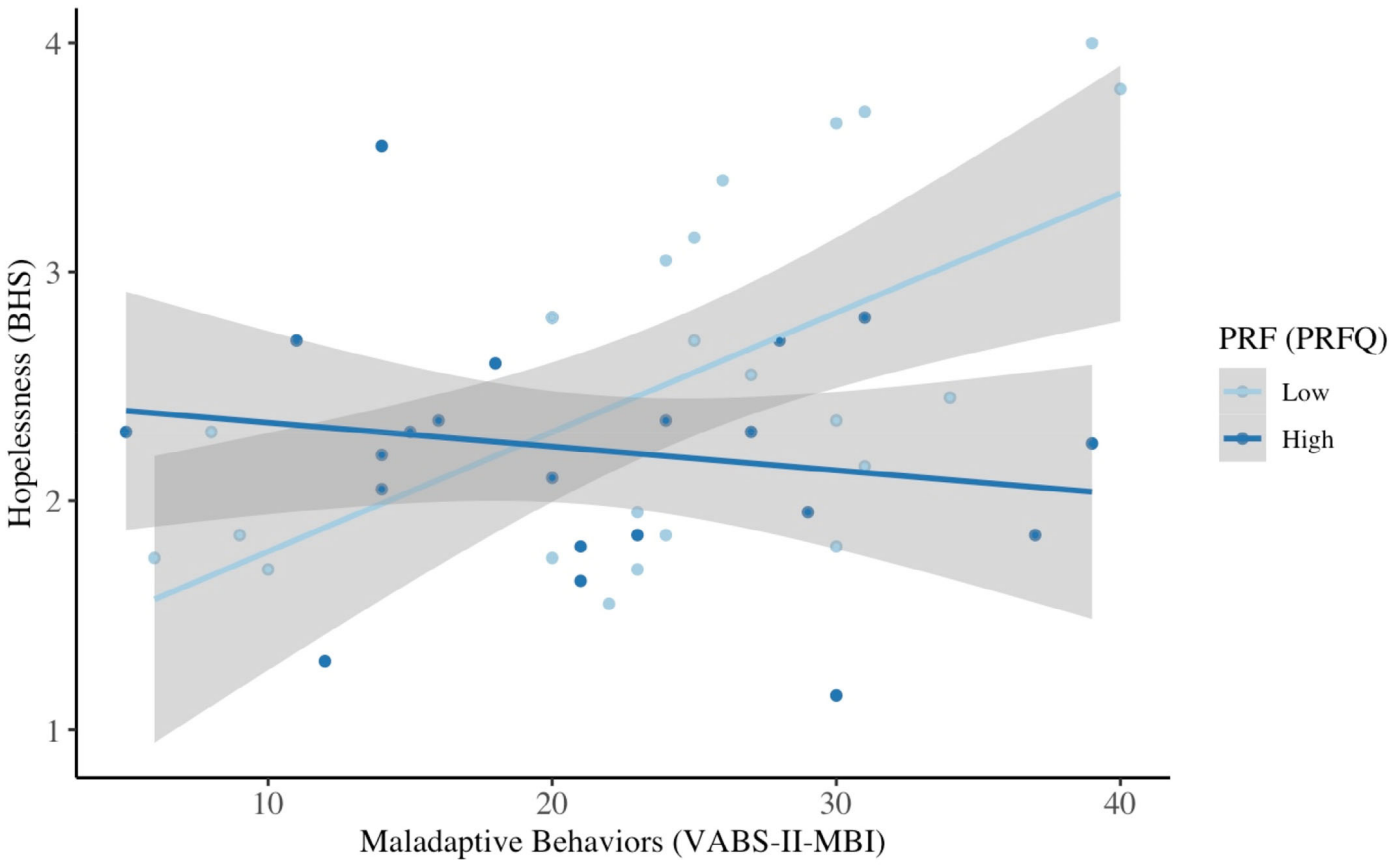
Moderation of PRFQ scores on the effect of VABS‐II‐MBI scores on BHS scores. VABS‐II‐MBI represents Vineland Adaptive Behavior Scale, second edition, Maladaptive Behavior Index. BHS represents Beck Hopelessness Scale. PRFQ represents Parental Reflective Functioning Questionnaire. PRF represents parental reflective functioning. High and low scores of PRF were calculated using a median split of PRFQ scores

PRF measured using self‐reported PRFQ scores significantly moderated the effect of total child maladaptive behavior on parental hopelessness (Figure 1, *F*(3,41) = 4.29, *p* < 0.05, *R* ^2^ = 0.24) such that the positive relationship between parental hopelessness and child maladaptive behaviors was strongest for parents low in PRF. See Figure 1 for moderation effects.

## DISCUSSION

The objective of this study was to examine the relationship between maladaptive behaviors in children with autism and parental hopelessness. As predicted, total child maladaptive behaviors significantly predicted parental hopelessness. In addition, our exploratory analyses revealed that the Other Maladaptive Behaviors subscale was driving the effect. Finally, as expected, the effect of total child maladaptive behaviors on parental hopelessness was moderated by PRF.

These findings are in line with previous studies that have found greater hopelessness in parents of children with maladaptive behaviors (Bradshaw et al., 2006; Padencheri & Russell, 2002). This could be due to the fact that the more parents are faced with their children's maladaptive behaviors, the harder it is for them to continue to hope that their children's behaviors will change in the future, ultimately leading to feelings of hopelessness. This is especially important to consider in parents of children with autism, as children with autism show elevated levels of maladaptive behaviors (Esteves et al., 2021; Mira et al., 2019), likely putting parents at greater risk for the development of hopelessness.

Of the three VABS‐II‐MBI subscales, the Other Maladaptive Behaviors subscale emerged as driving the effect on parental hopelessness. This is an interesting finding given that the Other subscale is unique to the VABS‐II‐MBI. Another study using the VABS‐II‐MBI (Hall & Graff, 2012) looked at the effect of maladaptive behaviors in children with autism on parental *stress* and found the internalizing subscale to drive the effect. In the context of parental *hopelessness*, however, the Other subscale may be more pronounced because, compared to the other two VABS‐II‐MBI subscales, it includes items that more directly relate to the symptoms of autism (e.g., “Has tics”; “Ignores or doesn't pay attention to others around him or her”). One possible explanation for these autism‐specific behaviors driving parental hopelessness could be that when parents experience their children's autism symptoms, they are reminded of their children's diagnosis and the difficulties it brings, eliciting feelings of hopelessness.

An important factor to consider related to the parent–child relationship, especially in families with children with autism, is PRF. Our results revealed that PRF moderated the effect of child maladaptive behaviors on hopelessness in parents. One way to think about this moderation effect is that parents high in PRF are able to recognize the changing nature of their children's behaviors, they understand that altering something about the present circumstances would change children's behaviors. In addition, parents high in PRF recognize the developmental aspects of their children's behaviors and that their behaviors will evolve as they mature. PRF also allows parents to understand that they can influence the behaviors of their children (Slade et al., 2005). Together, these reflections afforded by higher levels of PRF may help parents frame their children's behaviors as products of their children's current circumstances, developmental stage, and parental influences—all things that are constantly in flux.

These insights from higher level of PRF are directly related to one of the vulnerability factors for hopelessness: the attribution of negative life events to *stable* causes (Beck et al., 1974). Because parents high in PRF are able to understand their children's maladaptive behaviors as a result of unstable, fluctuating circumstances, they may be protected from feelings of hopelessness in response to their children's maladaptive behaviors. These findings are in line with those of a 2021 study by Khoshroo and Mousavi that found PRF to moderate the relationship between maternal depression and externalizing problems in their children.

The findings from this study have important clinical implications. Parental hopelessness, known to be elevated in parents of children with autism (Naci & Koletsi, 2021), has been linked to negative outcomes such as poor mental health in both parents and children (Ma et al., 2021). Understanding the effect of maladaptive behaviors in children with autism on parental hopelessness, and the role of PRF on this relationship, sheds light on ways to improve hopelessness in parents of children with autism. For example, if parents are able to reflect on the behaviors of their children with autism from the perspective of changing environmental influences, as in high PRF, they may be protected from feelings of hopelessness in response to their children's maladaptive behaviors.

Furthermore, the findings of this study raise the question of a possible effect of a broader autistic phenotype in parents of children with autism, which may make it more difficult for them to engage in PRF. It would be interesting for future studies to compare the level of PRF in parents of children with autism with parents of typically developing children or parents of children with other developmental disabilities.

While the current findings provide novel information regarding the relationship between maladaptive behaviors in children and parental hopelessness, this study has several limitations. First, our sample size was relatively modest. Second, the majority of our sample consisted of mothers of children with autism with low parent‐reported severity of symptoms, limiting the generalizability of the results. Third, our measure of child behavior was solely based on parent report, limiting a wider perspective on child behavior. Finally, because all of our measures were collected at a single time point, we were not able to draw causal inferences regarding the relationship between parental hopelessness and child behavior. Future studies would benefit from having a larger sample size that includes an equal representation of mothers and fathers and a wide range of autism severity. In addition, including multiple measures of child behavior and having a longitudinal design would provide future researchers with stronger grounds for causal inferences about the links among children's behavior, PRF, and parental hopelessness.

## CONFLICT OF INTEREST

The authors declare no conflict of interest.

## ETHICS STATEMENT

The study was approved by the university's Institutional Review Board and registered in the Clinical Trials database.

## Data Availability

The data that support the findings of this study are available from the corresponding author upon reasonable request.

## References

[aur2841-bib-0001] Beck, A. T. , Weissman, A. , Lester, D. , & Trexler, L. (1974). The measurement of pessimism: The hopelessness scale. Journal of Consulting and Clinical Psychology, 42(6), 861–865. 10.1037/h0037562 4436473

[aur2841-bib-0002] Bradshaw, C. P. , Glaser, B. A. , Calhoun, G. B. , & Bates, J. M. (2006). Beliefs and practices of the parents of violent and oppositional adolescents: An ecological perspective. The Journal of Primary Prevention, 27(3), 245–263. 10.1007/s10935-006-0030-3 16598659

[aur2841-bib-0003] Enav, Y. , Erhard‐Weiss, D. , Kopelman, M. , Samson, A. C. , Mehta, S. , Gross, J. J. , & Hardan, A. Y. (2019). A non randomized mentalization intervention for parents of children with autism. Autism Research, 12(7), 1077–1086. 10.1002/aur.2108 31002483

[aur2841-bib-0004] Enea, V. , & Rusu, D. M. (2020). Raising a child with autism Spectrum disorder: A systematic review of the literature investigating parenting stress. Journal of Mental Health Research in Intellectual Disabilities, 13(4), 283–321. 10.1080/19315864.2020.1822962

[aur2841-bib-0005] Esteves, J. , Perry, A. , Spiegel, R. , & Weiss, J. A. (2021). Occurrence and predictors of challenging behavior in youth with intellectual disability with or without autism. Journal of Mental Health Research in Intellectual Disabilities, 14(2), 189–201. 10.1080/19315864.2021.1874577

[aur2841-bib-0006] Fonagy, P. , Gergely, G. , Jurist, E. L. , & Target, M. (2006). Affect regulation, mentalization, and the development of the self. Karnac.

[aur2841-bib-0007] Gray, S. A. O. (2013). Maladaptive behavior. In F. R. Volkmar (Ed.), Encyclopedia of autism spectrum disorders. Springer. 10.1007/978-1-4419-1698-3_239

[aur2841-bib-0008] Gresham, F. M. , Lane, K. L. , Macmillan, D. L. , & Bocian, K. M. (1999). Social and academic profiles of externalizing and internalizing groups: Risk factors for emotional and behavioral disorders. Behavioral Disorders, 24(3), 231–245. 10.1177/019874299902400303

[aur2841-bib-0009] Hall, H. R. , & Graff, J. C. (2012). Maladaptive behaviors of children with autism: Parent support, stress, and coping. Issues in Comprehensive Pediatric Nursing, 35(3–4), 194–214. 10.3109/01460862.2012.734210 23140414

[aur2841-bib-0010] Kauten, R. , & Barry, C. T. (2020). Externalizing behavior. In V. Zeigler‐Hill & T. K. Shackelford (Eds.), Encyclopedia of personality and individual differences. Springer. 10.1007/978-3-319-24612-3_894

[aur2841-bib-0011] Khoshroo, S. , & Seyed Mousavi, P. S. (2021). Parental reflective functioning as a moderator for the relationship between maternal depression and child internalizing and externalizing problems. Child Psychiatry and Human Development, 53, 1319–1329. 10.1007/s10578-021-01214-6 34173125 PMC8231079

[aur2841-bib-0012] Luyten, P. , Mayes, L. C. , Nijssens, L. , & Fonagy, P. (2017). The parental reflective functioning questionnaire: Development and preliminary validation. PLoS One, 12(5), e0176218. 10.1371/journal.pone.0176218 28472162 PMC5417431

[aur2841-bib-0013] Ma, S. W. , Lai, S. , Yang, Y. Y. , Zhou, Z. , Yang, B. T. , Zheng, G. Z. Y. , Gao, J. , & Lu, L. (2021). Relationships between anxiety symptoms, hopelessness and suicidal ideation among parental caregivers of mandarin‐speaking children with speech impairment: The mediating effect of depressive symptoms. Frontiers in Psychiatry, 12, 498. 10.3389/fpsyt.2021.648885 PMC8110902 33986701

[aur2841-bib-0014] Mira, L. , Berenguer, C. , Roselló, B. , Baixauli, I. , & Miranda, A. (2019). Exploring the profiles of children with autism spectrum disorder: Association with family factors. International Journal of Developmental Disabilities, 68(1), 14–24. 10.1080/20473869.2019.1679459 35173960 PMC8843342

[aur2841-bib-0015] Naci, E. , & Koletsi, M. (2021). The relationship between cognitive distortions, hopelessness, and depression in parents of children diagnosed with autism spectrum disorder in Albania. Dialogues in Clinical Neuroscience & Mental Health, 4(2), 81–90. 10.26386/obrela.v4i2.160

[aur2841-bib-0016] Padencheri, S. , & Russell, P. S. S. (2002). Challenging Behaviours among children with intellectual disability. Journal of Learning Disabilities, 6(3), 253–261. 10.1177/1469004702006003035

[aur2841-bib-0017] Slade, A. (2009). Mentalizing the unmentalizable: Parenting children on the spectrum. Journal of Infant, Child, and Adolescent Psychotherapy, 8(1), 7–21. 10.1080/15289160802683054

[aur2841-bib-0018] Slade, A. , Grienenberger, J. , Bernbach, E. , Levy, D. , & Locker, A. (2005). Maternal reflective functioning, attachment, and the transmission gap: A preliminary study. Attachment & Human Development, 7(3), 283–298. 10.1080/14616730500245880 16210240

[aur2841-bib-0019] Sparrow, S. S. , Cicchetti, D. V. , & Balla, D. A. (2005). Vineland adaptive behavior scales (2nd ed.). American Guidance Service.

